# Zero-Dimensional Tellurium-Based Organic–Inorganic Hybrid Halide Single Crystal with Yellow-Orange Emission from Self-Trapped Excitons

**DOI:** 10.3390/nano14010046

**Published:** 2023-12-22

**Authors:** Xiangyan Yun, Jingheng Nie, Hanlin Hu, Haizhe Zhong, Denghui Xu, Yumeng Shi, Henan Li

**Affiliations:** 1 Department of Physics, Beijing Technology and Business University, Beijing 100048, China; 2 International Collaborative Laboratory of 2D Materials for Optoelectronics Science and Technology of Ministry of Education, Institute of Microscale Optoelectronics, Shenzhen University, Shenzhen 518060, China; 3 Guangdong Rare Earth Photofunctional Materials Engineering Technology Research Center, School of Chemistry and Environment, Jiaying University, Meizhou 514015, China; 4 Hoffman Institute of Advanced Materials, Shenzhen Polytechnic, Shenzhen 518060, China; 5 School of Electronics and Information Engineering, Shenzhen University, Shenzhen 518060, China; henan.li@szu.edu.cn

**Keywords:** Te-based, low dimensional, single crystal, hybrid halide, self-trapped exciton

## Abstract

Organic–inorganic hybrid halides and their analogs that exhibit efficient broadband emission from self-trapped excitons (STEs) offers an unique pathway towards realization of highly efficient white light sources for lighting applications. An appropriate dilution of ns^2^ ions into a halide host is essential to produce auxiliary emissions. However, the realization of ns^2^ cation-based halides phosphor that can be excited by blue light-emitting diode (LED) is still rarely reported. In this study, a zero-dimensional Te-based single crystal (C_8_H_20_N)_2_TeCl_6_ was synthesized, which exhibits a yellow-orange emission centered at 600 nm with a full width at half maximum of 130 nm upon excitation under 437 nm. Intense electron–phonon coupling was confirmed in the (C_8_H_20_N)_2_TeCl_6_ single crystal and the light emitting mechanism is comprehensively discussed. The results of this study are pertinent to the emissive mechanism of Te-based hybrid halides and can facilitate discovery of unidentified metal halides with broadband excitation features.

## 1. Introduction

Lead-free perovskite materials—which exhibit strong photoelectric conversion efficiency, exceptional photoluminescence quantum yield (PLQY), high light absorption coefficients, and excellent flexibility—are an active area of research in flexible displays, semiconductor high-energy lasers, X-ray scintillator, light-emitting diodes, and infrared detectors [1,2,3,4]. The general structural formula of perovskites has progressively developed from the primary AB(II)X_3_ to A_2_B(I)B(III)X_6_ (halide double perovskites), A_2_B(IV)X_6_ (vacancy-ordered perovskites), A_3_B(III)2X_9_ (halide triple perovskites), A_4_B(II)B(III)_2_X_12_ (halide quadruple perovskites) and other configurations [5,6,7]. A is monovalent organic or inorganic cations, such as CH_3_NH_3_^+^, NH_2_CHNH_2_^+^, Cs^+^, Rb^+^, K^+^; B(I), B(II), B(III) and B(IV), respectively, stand for metal cations with different valence states, such as Ag^+^, Cu^+^, Sn^2+^, Zn^2+^, Sb^3+^, In^3+^, Zr^4+^, Hf^4+^, Te^4+^, and Sn^4+^; X represents monovalent halide anion, such as Cl^−^, I^−^, Br^−^ or mixed halide anions [8,9]. Moreover, perovskites and perovskite derivatives can be classified into three-, two-, one-, and zero-dimensional (0D) at the molecular level [10]. Low-dimensional metal halide materials have large forbidden bands and high exciton binding energies, because of their remarkable crystal and electronic structures [11,12]. Introducing organic molecules leads to extraordinary possibilities in structure and performance research of metal halides with various dimensions [13]. Therefore, developing organic–inorganic hybrid, lead-free, low-dimensional metal halides is important for expanding functional applications.

Presently, the exceptional photoelectric performance of organic–inorganic hybrid, lead-free, low-dimensional metal halides is particularly influenced by B-site metal cations [14]. Metal cations containing the outermost electron arrangement as ns^2^ lone pair electrons are called ns^2^ ions. 5S^2^ ions (Sn^2+^, Sb^3+^, Te^4+^) and 6S^2^ ions (Bi^3+^, Pb^2+^) can exhibit their lone pair electron transition characteristics during photoexcitation. Metal halide materials containing ns^2^ cations have advantages compared with analogous materials that contain other cations, in terms of outstanding self-trapped exciton (STE) emission and other optoelectronic characteristics [15,16,17,18]. For example, the Zhao group reported that (C_10_H_22_N)_2_SbBr_5_ and (C_10_H_22_N)_3_Sb_2_Br_9_ exhibit orange emission with maxima at 640 and 660 nm, respectively, which originates from STE emission of the Sb^3+^ cation [19]. Nevertheless, currently, the widely investigated Sb-based halides cannot be excited with blue light, which also occurs in, e.g., Bi- or Ge-based halides [20,21]. Mn-based halides can be excited with blue light; however, the luminescence mechanism of Mn-based halides is attributable to the *d*-*d* transition of Mn ions, which leads to their lack of bright STE broad-spectrum emission [22,23,24]. In that vein, an immense challenge in STE emitters is finding an STE emitter with ultra-broadband excitation involving blue light regions.

Significantly, tetravalent tellurium ion with a lone-pair 5s^2^ electronic configuration was found to be an ion accompanied by distinctive STE emission. And the ultra-wide absorption band present in Te-based or Te-doped metal halides may cause them to be excited by blue light [25]. Recently, some progress has been achieved in all-inorganic metal halides-doped Te^4+^ ions with STE emission that can be excited by blue light. For example, Zhang et al. attributed the luminescence of Te^4+^-doped Rb_2_SnCl_6_ that can be excited by blue light to the STE emission of the [TeCl_6_]^2−^ octahedron [26]. Li et al. developed 0D Sc(III)-based Te^4+^-doped Rb_2_ScCl_5_·H_2_O single crystals with a broadband orange STE emission (650 nm) that can be excited by light in the range of 240 nm to 440 nm [27]. Compared with inorganic metal halides doped with Te^4+^ ions, the STE emission research on Te-based organic–inorganic hybrid metal halides usually show limited light emission efficiency. For instance, Mirochnik et al. discovered the broadband emission of ((CH_3_)_4_N)_2_TeCl_6_ halide at 77 K, with poor luminescence at room temperature [28]. For the time being, there are few Te-based organic–inorganic hybrid metal halides that can exhibit obvious STE emission at room temperature, although some Te-based halides can exhibit relatively high luminescence intensity at low temperatures. Therefore, there is an urgent need for new low-dimensional Te-based metal halides that exhibit STE emission at room temperature.

Inspired by the low dimensionality and configuration of metal halides, the authors of this paper designed and synthesized an organic–inorganic hybrid, lead-free, 0D (C_8_H_20_N)_2_TeCl_6_ single crystal. The structural details, optical and decay lifetime characteristics at room temperature, as well as temperature-dependent luminescence of this single crystal are discussed in detail. At room temperature, (C_8_H_20_N)_2_TeCl_6_ single crystals exhibit a broad-band STE emission (λ_em_ = 600 nm) with a full width at half maximum (FWHM) of 130 nm under 437 nm excitation. Temperature-dependent data also demonstrate that the yellow-orange emission is attributable to archetypal STE emission.

## 2. Experimental Section

### 2.1. Materials 

Tetraethylammonium chloride (C_8_H_20_NCl, 98%, Aladdin, Shanghai, China), tellurium tetrachloride (TeCl_4_, 99%, Meryer, Shanghai, China), anhydrous ethanol (C_2_H_6_O, AR, Macklin, Shanghai, China), diethyl ether (C_4_H_10_O, AR, Sinopharm Chemical Reagent Co., Ltd., Shanghai, China) and hydrochloric acid (HCl, Sigma-Aldrich, Shanghai, China) were used as received. During the process of synthesizing (C_8_H_20_N)_2_TeCl_6_ single crystal, TeCl_4_ materials can also be replaced with tellurium dioxide (TeO_2_, 99.99%, Bide Pharmatech Co., Ltd., Shanghai, China).

### 2.2. Synthesis of (C_8_H_20_N)_2_TeCl_6_ Single Crystal

Anti-solvent evaporation and traditional solvothermal methods can be used to synthesize single crystals. Regarding the traditional solvothermal method, the solvent is HCl. C_8_H_20_NCl (0.6628 g), TeCl_4_ (0.5388 g) and 16 mL hydrochloric acid were loaded into a 25 mL tetrafluoroethylene reactor, heated at 120 °C for 8 h, and cooled to room temperature within 16 h. Figure S1A shows the schematic process of synthesizing (C_8_H_20_N)_2_TeCl_6_ single crystals using solvothermal method. It is worth noting that after the reaction completes, yellowish-green single crystals can be obtained in the hydrochloric acid liquid. Then, the prepared single crystals are transferred from the tetrafluoroethylene reactor to a culture dish. In order to remove the hydrochloric acid attached to the surface of the (C_8_H_20_N)_2_TeCl_6_ single crystals, an appropriate amount of anhydrous ethanol was added to the culture dish to rinse the prepared single crystals. The above flushing process is repeated for at least three times. Finally, the anhydrous ethanol in this culture dish was removed by a dropper and placed in an oven at 65 °C for 20 min of drying treatment. In addition, this experimental process can also replace 0.5388 g of TeCl_4_ with 0.3192 g of TeO_2_, while keeping most of the remaining experimental requirements unchanged. When washing (C_8_H_20_N)_2_TeCl_6_ single crystals prepared using TeO_2_ as raw material, it is necessary to replace anhydrous ethanol with isopropanol. However, it should be noted that the single crystal luminescence performance synthesized using TeCl_4_ is superior to that synthesized using TeO_2_. The luminescence characteristics detected in this article are the properties of single crystals prepared using TeCl_4_. Regarding anti-solvent evaporation, first, 0.002 mol C_8_H_20_NCl (0.3314 g) and 0.001 mol TeCl_4_ (0.2694 g) were dissolved in a single solvent (1.5 mL C_2_H_6_O at 65 °C) to form a clear precursor solution (Figure S1B). Then, the as-formed precursor solution was transferred to an unsealed 10 mL glass bottle, the mouth of this 10 mL glass bottle was sealed with plastic film, and sharp needles were used to puncture several breathable openings. Finally, this small glass bottle was placed into a large sealed weighing bottle with diethyl ether reagent at the bottom. Within 3 d at room temperature, the yellowish-green crystals formed. The (C_8_H_20_N)_2_TeCl_6_ single crystals synthesized by the anti-solvent evaporation method were washed with anhydrous ethanol. The drying conditions were consistent with those used in the traditional solvothermal method above.

### 2.3. Characterization 

Powder XRD measurements of (C_8_H_20_N)_2_TeCl_6_ powder were conducted using an X-ray diffractometer (D2 Phaser) with Cu-Kα radiation (λ = 1.5418 Å, 40 kV, 20 mA). The data used for Powder XRD analysis (2*θ* range 5–60°) was collected in a step-scanning mode with a step size of 0.02° and 0.5 s counting time per step. The single crystal XRD measurements were performed with a Bruker D8 Venture diffractometer. (C_8_H_20_N)_2_TeCl_6_ crystals were maintained at 193.15 K during data collection. In Olex2, the structure was solved with the olex2.solve structure solution program using charge-flipping, and refined with the XL refinement package using least-squares minimization [29,30,31]. The absorption spectra were collected using a UV-3600i Plus UV–visible–NIR spectrophotometer (Shimadzu, Kyoto, Japan), in which BaSO_4_ commercial backing plate was used as the reference standard. The photoluminescence excitation (PLE) and photoluminescence (PL) patterns were recorded with FLUOROMAX_PLUS fluorescence spectrometer (HORIBA Scientific, Kyoto, Japan). The correlated color temperature (CCT) and color purity of (C_8_H_20_N)_2_TeCl_6_ powder were calculated using ColorCalculator v7.77 software. The color purity was calculated based on $Color\;purity=\sqrt{\frac{{\left(x_{r}-x_{i}\right)}^{2}+{\left(y_{r}-y_{i}\right)}^{2}}{{\left(x_{m}-x_{i}\right)}^{2}+{\left(y_{m}-y_{i}\right)}^{2}}\times 100\%}$ equation, in which (*x_i_*, *y_i_*) was the standard white light coordinate, (*x_m_*, *y_m_*) represented the Commission Internationale de I’Eclairage (CIE) coordinate of the dominant wavelength, and (*x_r_*, *y_r_*) was the CIE coordinate of (C_8_H_20_N)_2_TeCl_6_ halide. The PLQY pattern was recorded with an FS5 spectrofluorometer (Edinburgh integrated steady-state transient fluorescence spectrometer) at room temperature. The decay lifetimes were measured using an FS5 instrument equipped with a 405 nm (Edinburgh Instruments, Livingston, Scotland, UK; EPL-405 picosecond pulsed diode laser, 5 mW) laser. 

The time width of the 405 nm laser used for the dynamics of emission decays was 5 µs window, and the single-photon counting capability was 5000 counts (1024 channels). Temperature-dependent PL spectroscopic measurements were conducted using an FS5 equipped with a temperature precision controller and liquid nitrogen input equipment (Beijing Physike Technology Co., Ltd., Beijing, China).

## 3. Results and Discussion

**Figure 1:**
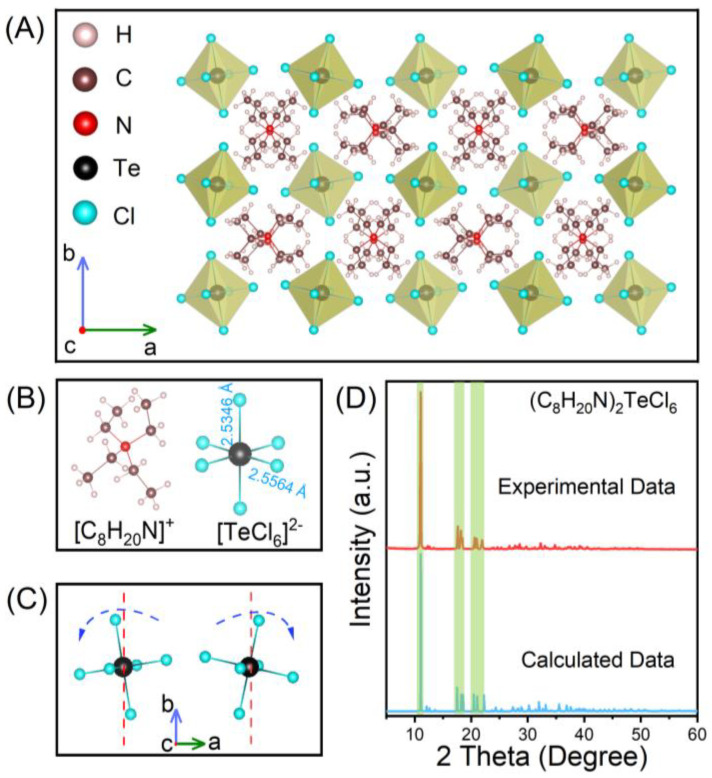
(**A**) Structural diagram of (C_8_H_20_N)_2_TeCl_6_ single crystals. (**B**) The structure of the individual [C_8_H_20_N]^+^ and [TeCl_6_]^2−^. (**C**) [TeCl_6_]^2−^ octahedral distortion diagram. (**D**) XRD pattern of (C_8_H_20_N)_2_TeCl_6_.

As-grown (C_8_H_20_N)_2_TeCl_6_ single crystals were synthesized using the traditional solvothermal method, and single crystal X-ray diffraction (SCXRD) was used to confirm its structure. The crystal structure of (C_8_H_20_N)_2_TeCl_6_ exhibited a monoclinic space group *P2_1_/c*; the cell parameters were *a* = 14.0511 Å, *b* = 14.6234(15) Å, *c* = 13.0285(15) Å, and *V* = 2677.0(5) Å^3^. The crystallographic information file (CIF) of (C_8_H_20_N)_2_TeCl_6_ single crystal has been uploaded to the Cambridge Crystallographic Data Centre (CCDC#2304048). Furthermore, Tables S1–S7 (Supporting Information) show the remaining essential crystallographic data as well as relevant parameter analysis, respectively. As shown in Figure 1A, (C_8_H_20_N)_2_TeCl_6_ exhibited a 0D structure with units of [TeCl_6_]^2−^ octahedral, and each isolated [TeCl_6_]^2−^ octahedron was surrounded by extensive organic molecular (C_8_H_20_N)^+^ units [32,33]. Figure 1B shows that each [TeCl_6_]^2−^ octahedron contains two Te-Cl bond distances (2.5346 Å and 2.5564 Å) and two Cl-Te-Cl bond angles (89.371° and 90.629°). From the same perspective, the phenomenon of two [TeCl_6_]^2−^ octahedra twisting in opposite directions can be observed (Figure 1C). The simulated SCXRD pattern of (C_8_H_20_N)_2_TeCl_6_ is highly consistent with the powder XRD pattern (Figure 1D), which indicates that the powder prepared by grinding exhibited an identical structure as the single crystal [34]. Figure S2 shows a locally enlarged XRD pattern, which also demonstrates good consistency between the experimental data and the calculated data.

Regarding Pb-free metal halide luminescent systems, the luminescent behavior of ns^2^ metal ions as visible and near-infrared (NIR) luminescent centers is an active area of research. Te^4+^, Sb^3+^, Sn^2+^ and Bi^3+^ ions exhibit analogous chemical properties and electronic configurations as significant ions [15,35]. The Te^4+^ ion containing 5s^2^ lone pair electrons has a similar ground state electronic structure to the Sb^3+^ ion that has been extensively studied in the field of metal halide luminescence. Figure S3A shows a schematic of the excitation state of Te^4+^ in metal halides. The excited states (sp) split into two branches (^1^P_1_ and ^3^P_n_) under the influence of spin–orbit coupling (SOC); the excited-state splitting phenomena of ^1^P_1_ and ^3^P_1_ follows the order of energy from low to high, which is usually attributed to the lattice symmetry breaking caused by the Jahn–Teller effect [36]. Several previously reported papers (Cs_2_SnCl_6_:Te^4+^; Cs_2_ZrCl_6_:Te^4+^) have confirmed the level (^1^P_1_ and ^3^P_1_) splitting phenomenon of Te^4+^ ions [37,38]. ^1^S_0_–^1^P_1_, ^1^S_0_–^3^P_2_, and ^1^S_0_–^3^P_1_ transitions are allowed, vibration-induced, and spin–orbit-allowed, respectively. The spin–orbit-allowed behavior can derive from the intense effect of SOC by tellurium atoms [38,39]. Deformation of octahedra is common in [TeCl_6_]^2−^ octahedra that are susceptible to the Jahn–Teller effect (Figure S3B). The vibrational mode of [TeCl_6_]^2−^ octahedra in (C_8_H_20_N)_2_TeCl_6_ belongs to *v*2 mode [35]. 

**Figure 2:**
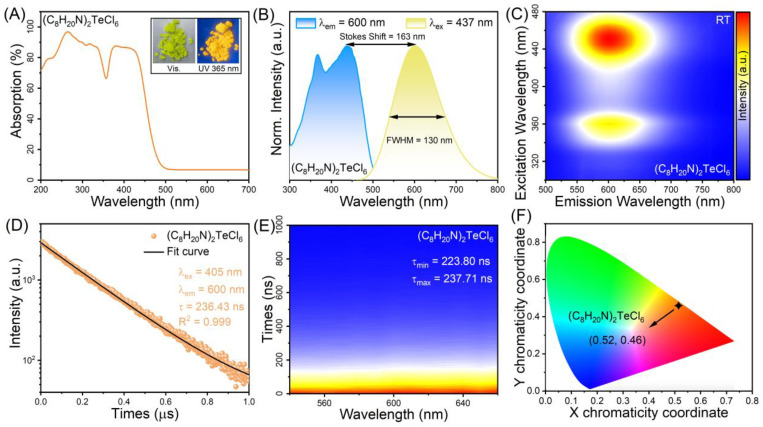
(**A**) Absorption spectra of (C_8_H_20_N)_2_TeCl_6_. The inset shows the optical images of (C_8_H_20_N)_2_TeCl_6_ under the illumination of daylight and 365 nm UV light. (**B**) PLE and PL spectra of (C_8_H_20_N)_2_TeCl_6_ at RT. (**C**) Pseudo color map of PL/PLE of (C_8_H_20_N)_2_TeCl_6_. (**D**) Emission wavelength scanning decay of (C_8_H_20_N)_2_TeCl_6_ excited by 405 nm. (**E**) Emission wavelength scanning decay of (C_8_H_20_N)_2_TeCl_6_ excited by 405 nm. (**F**) CIE color coordinate of (C_8_H_20_N)_2_TeCl_6_ sample.

Figure 2A shows the UV–visible absorption spectrum of (C_8_H_20_N)_2_TeCl_6_ halide. The absorption curve of (C_8_H_20_N)_2_TeCl_6_ halide exhibits an ultra-wide absorption band in the wavelength range of 200–500 nm, which is similar to reports for (C_13_H_22_N)_2_TeCl_6_ and (C_20_H_20_N)_2_TeCl_6_ [40,41]. The ultrabroad absorption bands are attributable to the dipole-allowed, vibration-allowed, and spin–orbit-allowed transitions of Te^4+^ ions. And the inset shows the optical images of (C_8_H_20_N)_2_TeCl_6_ under the illumination of daylight and 365 nm UV light. (C_8_H_20_N)_2_TeCl_6_ single crystal appears yellowish-green and transparent in daylight; however, when exposed to UV light at 365-nm, it displays yellow-orange fluorescence. In addition, the same emission occurs in the (C_8_H_20_N)_2_TeCl_6_ powder. Figure 2B shows the normalized room-temperature PL emission and excitation spectra of (C_8_H_20_N)_2_TeCl_6_. The PLE spectrum (monitored at 600 nm) consists of an ultrabroad band in the UV and blue regions with a peak at 437 nm, indicating that this halide could be excited with available blue light-emitting diode chips. Upon excitation at 437 nm, (C_8_H_20_N)_2_TeCl_6_ exhibited a strong-broad yellow-orange emission ranging from 450 to 800 nm, with a FWHM of 130 nm and a peak at 600 nm. In addition, there was a large Stokes shift of 163 nm. Figure 2C shows the pseudo-color map of a PL/PLE of (C_8_H_20_N)_2_TeCl_6_ sample at room temperature, in which the characteristics of emission spectra under variable excitation and STE emission characteristics of Te^4+^ ions can have a precise correspondence [42]. To gain a deeper understanding of the luminous mechanism in the (C_8_H_20_N)_2_TeCl_6_ system, further information is obtained from wavelength-scanning time-correlated decay experiments. Under 405 nm laser excitation, the lifetime monitored at 600 nm in (C_8_H_20_N)_2_TeCl_6_ can be fitted using a single exponential equation: *I* = *A*e^−*t*/*τ*^, in which *I* is the PL intensity, *A* refers to the constant, and *τ* represents the fitting decay time. The fitting lifetime of (C_8_H_20_N)_2_TeCl_6_ halide was 236.43 ns, and the value of R^2^ (R^2^ = 0.999) indicates that this fitting is reliable (Figure 2D). The decay-mapping and fitting results are shown in Figure 2E and Table S8 (Supporting Information). The emission peaks located from 540 nm to 660 nm represent similar fitting lifetimes, indicating that there was only one PL component. The multiple lifetimes fitted by (C_8_H_20_N)_2_TeCl_6_ sample are similar to the previously reported lifetime characteristics of STE luminescence behavior related to Te^4+^ ion doping (Rb_2_ZrCl_6_:Te^4+^, 235.62 ns; Cs_2_ZnCl_4_:Te^4+^, 53.2 ns) or Te-based materials ((Ph_3_S)_2_TeCl_6_, 42.7 ns) [35,42,43]. The lifetimes of hundreds of nanoseconds are attributable to STE emission of [TeCl_6_]^2−^ octahedrons. The CIE coordinate of (C_8_H_20_N)_2_TeCl_6_ halide is (0.52, 0.46) with a CCT of 2376 K and a color purity of 95.2% (Figure 2F), which is higher than that of Cs_2_SnCl_6_:Te^4+^ (84.69%) [44]. Regarding Te-based halides, a constraint is that the PLQY at room temperature is low, which can cause STE luminescence emission that is insubstantial to the unaided eye. Here, the PLQY at room temperature was 10.12% (Figure S4).

**Figure 3:**
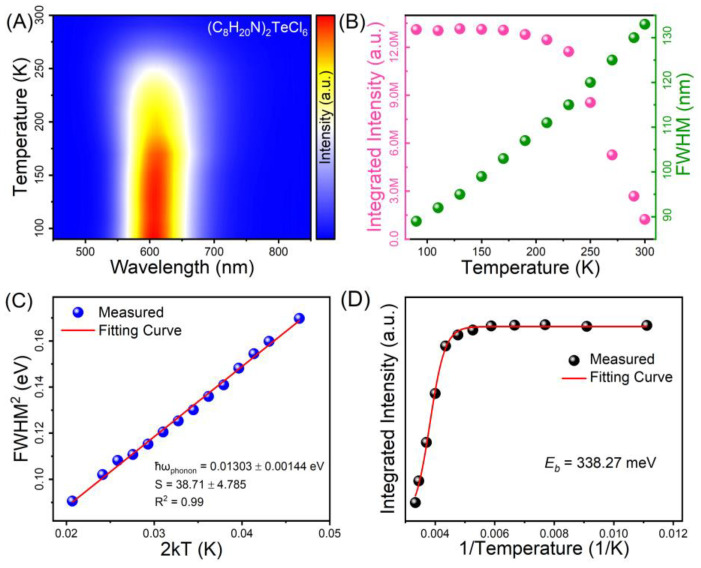
(**A**) Temperature-dependent PL spectrum of (C_8_H_20_N)_2_TeCl_6_. (**B**) Integrated intensity and FWHM as functions of temperature. (**C**) FWHM^2^ as functions of 2 kT. (**D**) Integrated PL intensity as functions of 1/T.

**Figure 4:**
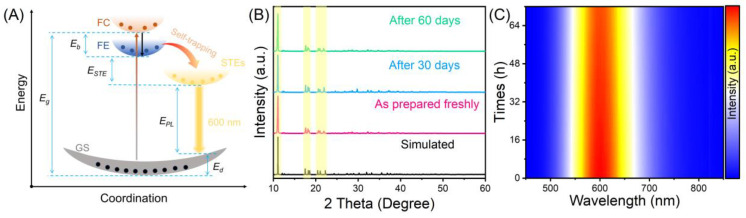
(**A**) Configuration coordinate diagram demonstrating the photophysical process in (C_8_H_20_N)_2_TeCl_6_. (**B**) PXRD patterns of (C_8_H_20_N)_2_TeCl_6_ stored in the atmosphere for 60 d. (**C**) The PL map of (C_8_H_20_N)_2_TeCl_6_ sample under different blue light excitation times.

To obtain deep insights into the mechanism of STE emission of Te-based halides, temperature-dependent PL spectroscopic measurements of (C_8_H_20_N)_2_TeCl_6_ halide were implemented in the range of 90 K to 300 K. Figure 3A shows the temperature-dependent PL spectra of (C_8_H_20_N)_2_TeCl_6_. The test temperature was negatively correlated with the PL intensity. As the test temperature continued to increase from cryogenic to room temperature, an inconspicuous blue shift of the emission band was observed, which originates from electron–phonon coupling interactions and thermal expansion of the metal halide lattice [45]. According to the currently recognized theory of STE luminescence in metal halides, the STE emissions are affected by the strength of exciton–phonon coupling. Figure 3B shows a plot of the FWHM (obtained using Gaussian fitting) and integrated PL intensity against the temperature. Conventional theories use the Huang–Rhys factor (*S*), dimensionless, to characterize the strength of electron–phonon coupling, which exhibits a close correlation with the FWHM. The Huang–Rhys factor can be calculated using the fitting formula [46]:

(1) $$\mathrm{FWHM}=2.36\sqrt{S}\hbar \omega_{phonon}\sqrt{coth\frac{\hbar \omega_{phonon}}{2k_{B}T}}$$

where *ħω_phonon_*, *T*, and *k_B_* represent the phonon frequency, practical temperature, and the Boltzmann constant, respectively. Through multiple derivations and simplifications of Formula (1), Figure 3C shows the functional relationship between FWHM^2^ vs. 2*kT* as well as the linear fitting line (red line). Regarding the (C_8_H_20_N)_2_TeCl_6_ halide, the phonon frequency and Huang–Rhys factor were ca. 0.01303 eV, and 38.71, respectively. In general, there is a certain correlation between the *S* value and the ease of STE formation. For STE emissions of metal halides, the appropriate value of *S* is generally between 10 and 40 [47]. For instance, pure inorganic Sb^3+^-doped Rb_4_CdCl_6_ sample with typical STE emission has an *S* of 34.94, which is extremely similar to the 38.71 of (C_8_H_20_N)_2_TeCl_6_ halide [48]. The Huang–Rhys factor of (C_8_H_20_N)_2_TeCl_6_ halide is evidently higher than the previously reported halides of Cs_2_Sn_0.89_Te_0.11_Cl_6_ (16.0), (CH_3_CH_2_CH_2_)_4_NCuCl_2_ (16.4), and Sb^3+^-doped Cs_3_Cd_2_Cl_7_ (30.06) with emblematic STE emissions, indicative of intense exciton–phonon coupling and Jahn–Teller distortion in (C_8_H_20_N)_2_TeCl_6_ [48,49,50]. Moreover, according to the STE emission calculation formula (*E_PL_* = *E_g_* − *E_b_* − *E_STE_* − *E_d_*), the exciton binding energy (*E_b_*) is a pertinent physical parameter that is used to deduce STE emission. Figure 3D shows the temperature-dependent integrated PL intensity; the *E_b_* values were determined using the Arrhenius expression:

(2) $$I\left(T\right)=I_{0}/\left(1+Aexp^{-E_{b}/k_{B}T}\right)$$

where *I*(*T*) and *I*_0_ represent the integrated intensity at the actual measurement temperature and the integrated intensity at 0 K, respectively. The *E_b_* of (C_8_H_20_N)_2_TeCl_6_ was ca. 338.27 meV, which is comparable with the previously reported all-inorganic low-dimensional metal halides of Rb_2_ScCl_5_·H_2_O:Te^4+^ (384 meV), and CsCu_2_I_3_ (346 meV) [27]. The fitted exciton binding energy and Huang–Rhys factor can both prove that this yellow-orange broadband emission at 600 nm in (C_8_H_20_N)_2_TeCl_6_ halide is attributed to STEs of [TeCl_6_]^2−^ octahedral [36,37]. Overall, the luminescence mechanism for (C_8_H_20_N)_2_TeCl_6_ halide (Figure 4A) can be summarized as follows: UV or blue-light excitation leads to the electron excitation from the ^1^S_0_ state to the excited states of Te–Cl. Then, the free carriers (FCs) become free excitons (FEs) through coulombic interactions. In addition, the low-dimensional (0D) structure of the (C_8_H_20_N)_2_TeCl_6_ halide is beneficial for promoting the presence of a soft lattice in this single crystal, thus easily forming STE emission. FEs are subjected to intense exciton–phonon interactions, resulting in lattice distortion of the [TeCl_6_]^2−^ octahedral and trapping in the STE state. Therefore, FEs ultimately fall into the newly formed STE state, leading to yellow-orange STE emission. Moreover, as shown in Figure 4B, the PXRD peak pattern of the (C_8_H_20_N)_2_TeCl_6_ sample under long-term air exposure conditions exhibit significant consistency, with several major PXRD peaks corresponding to each other in the highlighted yellow area. And the PL intensity of the (C_8_H_20_N)_2_TeCl_6_ powder after continuous exposure to 437 nm blue light for 70 h is not significantly different from the original PL intensity (Figure 4C). Hence, the above XRD and spectral results indicate that the as-synthesized (C_8_H_20_N)_2_TeCl_6_ samples have a reliable stability.

## 4. Conclusions

In summary, we successfully synthesized a 0D (C_8_H_20_N)_2_TeCl_6_ single crystal through various methods and investigated the STE mechanism of broadband luminescence. The crystal structure of (C_8_H_20_N)_2_TeCl_6_ was identified in the space group *P2_1_/c* and comprised [TeCl_6_]^2−^ octahedral and charge-balancing C_8_H_20_N^+^ cations. (C_8_H_20_N)_2_TeCl_6_ single crystals exhibited a yellow-orange emission (≈600 nm) with a large FWHM of 130 nm. And it exhibits a unique ultrabroad excitation band containing a blue excitation region. The color purity of (C_8_H_20_N)_2_TeCl_6_ halide is 95.2%. The temperature-dependent optical properties demonstrate that the Huang–Rhys factor and exciton binding energy were 38.71 and 338.27 meV, respectively, indicative of strong electron–phonon coupling. Therefore, we attributed the origin of the yellow-orange emission band to STE emission of [TeCl_6_]^2−^ octahedral. Our achievement in (C_8_H_20_N)_2_TeCl_6_ single crystals offers a strategy for designing and synthesizing Pb-free, low-dimensional, luminescent metal halides with broad blue-light excitation.

## Data Availability

The data presented in this study are available on request from the corresponding author.

## Supplementary Materials

The following supporting information can be downloaded at https://www.mdpi.com/article/10.3390/nano14010046/s1, Figure S1: Diagram of synthesis of (C_8_H_20_N)_2_TeCl_6_; Figure S2: PXRD patterns of (C_8_H_20_N)_2_TeCl_6_; Figure S3: Scheme of the energy level of (C_8_H_20_N)_2_TeCl_6_ and the *v*2 vibration mode of [TeCl_6_]^2−^ octahedra; Figure S4: PLQY of (C_8_H_20_N)_2_TeCl_6_; Tables S1−S7: crystallographic data; Table S8: PL lifetimes of (C_8_H_20_N)_2_TeCl_6_.
