# Supplementary material for: Zero-Dimensional Tellurium-Based Organic–Inorganic Hybrid Halide Single Crystal with Yellow-Orange Emission from Self-Trapped Excitons
Source: Nanomaterials (Basel). 2023 Dec 22;14(1):46. doi: 10.3390/nano14010046 (PMC10780417; doi:10.3390/nano14010046)
Supplement: Supplementary file 1 [file nanomaterials-14-00046-s001.zip › nanomaterials-2753595-supplementary.pdf]

nanomaterials

Supporting Information

Zero-Dimensional Tellurium-Based Organic–Inorganic Hybrid Halide Single Crystal with Yellow-Orange Emission from Self-Trapped Excitons

Xiangyan Yun [1,2], Jingheng Nie [3], Hanlin Hu [4], Haizhe Zhong [2], Denghui Xu [1,*], Yumeng Shi [5,*] and Henan Li [5]

1 Department of Physics, Beijing Technology and Business University, Beijing 100048, China; 2250493009@email.szu.edu.cn
2 International Collaborative Laboratory of 2D Materials for Optoelectronics Science and Technology of Ministry of Education, Institute of Microscale Optoelectronics, Shenzhen University, Shenzhen 518060, China; haizhe.zhong@szu.edu.cn
3 Guangdong Rare Earth Photofunctional Materials Engineering Technology Research Center, School of Chemistry and Environment, Jiaying University, Meizhou 514015, China; niejh@jyu.edu.cn
4 Hoffman Institute of Advanced Materials, Shenzhen Polytechnic, Shenzhen 518060, China; hanlinhu@szpt.edu.cn
5 School of Electronics and Information Engineering, Shenzhen University, Shenzhen 518060, China; henan.li@szu.edu.cn
* Correspondence: xudh@btbu.edu.cn (D.X.); yumeng.shi@szu.edu.cn (Y.S.)

Table S1. Crystal data and structure refinement for $(C_8H_{20}N)_2TeCl_6$ at 193.15 K.

Empirical formula	$C_{16}H_{40}Cl_6N_2Te$
Formula weight	457.24
Temperature/K	193.15
Crystal system	monoclinic
Space group	$P2_1/c$
a/Å	14.0511(15)
b/Å	14.6234(15)
c/Å	13.0285(15)
α/°	90
β/°	90.05(2)
γ/°	90
Volume/Å^3	2677.0(5)
Z	4
ρcalcg/cm^3	1.491
Radiation	MoKα (λ = 0.71073)
2θ range for data collection/°	4.02 to 67.432
Index ranges	$-21 \leq h \leq 20$, $-21 \leq k \leq 21$, $-18 \leq l \leq 20$
Reflections collected	83186
Independent reflections	9315 [R_{int} = 0.0888, R_{sigma} = 0.0572]
Data/restraints/parameters	9315/0/201
Goodness-of-fit on F^2	1.095
Final R indexes [I>=2σ (I)]	R_1 = 0.0354, wR_2 = 0.0789
Final R indexes [all data]	R_1 = 0.0845, wR_2 = 0.0959
Largest diff. peak/hole / e Å^{-3}	0.62/-2.81

Table S2. Fractional Atomic Coordinates (×10[4]) and Equivalent Isotropic Displacement Parameters (Å^2 × 10^3) for $(C_8H_{20}N)_2TeCl_6$ at 193.15 K. U_{eq} is defined as 1/3 of the trace of the orthogonalized U_{IJ} tensor.

Atom.	*x*	*y*	*z*	U_{eq}
N2	7486.4(11)	2427.4(13)	2506.2(12)	29.3(4)
C15	7812.7(16)	1835.9(16)	1609.6(17)	37.7(5)
C11	7156.1(16)	1851.6(15)	3416.3(17)	37.1(5)
C9	6661.9(16)	3017.7(16)	2154.5(18)	38.8(5)
C13	8319.0(16)	3010.9(15)	2846.6(17)	37.4(5)
C16	8548.6(19)	1118.5(18)	1868(2)	52.2(7)
C14	8079(2)	3713.8(17)	3664.8(19)	51.2(7)
C12	6395.3(19)	1156.5(18)	3176(2)	54.2(7)
C10	6917(2)	3718.8(18)	1336.1(19)	51.4(7)
N1	2421.5(12)	2387.6(13)	2346.1(13)	33.7(4)
C2	2057.1(16)	2277.6(15)	1256.6(16)	36.0(5)
C7	3048.9(17)	3233.1(16)	2367.5(19)	43.7(6)
C4	1617.2(17)	2510.2(15)	3118.7(18)	40.1(5)
C1	1333.1(18)	1523.0(16)	1106.7(19)	44.2(6)
C3	947.6(18)	3288.2(17)	2901(2)	49.3(6)
C5	2967(2)	1536.1(16)	2629.1(18)	47.5(6)
C6	3464(2)	1564(2)	3662(2)	57.0(8)
C8	3941.2(19)	3185(2)	1718(2)	66.8(9)
Te2	10000	5000	0	26.14(5)
Te1	5000	5000	5000	28.02(5)
Cl5	9464.6(4)	5064.5(3)	1862.8(4)	37.26(12)
Cl2	5386.1(5)	3299.3(4)	5071.9(5)	45.52(14)
Cl3	3259.4	4630.7	4670.4	44
Cl1	5352.4	4953.9	3092	40
Cl4	8282.2	5260.7	-518.1	39
Cl6	9698.1	3276.1	-9.8	38

Table S3. Anisotropic Displacement Parameters (Å^2 × 10^3) for $(C_8H_{20}N)_2TeCl_6$. The Anisotropic displacement factor exponent takes the form: $-2\pi^2[h^2a^{*2}U_{11} + 2hka^*b^*U_{12} + \ldots]$.

Atom	Atom	Length/Å	Atom	Atom	Length/Å	
N2	29.0(9)	31.4(9)	27.4(9)	-0.6(7)	-0.5(7)	1.1(7)
C15	40.3(13)	38.1(12)	34.7(12)	-7.1(9)	2.1(10)	4.6(10)
C11	38.7(12)	37.1(12)	35.5(13)	5.3(9)	2.0(10)	-3.3(10)
C9	35.8(12)	42.7(12)	37.9(13)	-1.0(10)	-3.7(10)	11.0(10)
C13	36.6(12)	37.4(11)	38.1(13)	1.0(10)	-3.0(10)	-7.6(10)
C16	50.4(15)	50.6(14)	55.7(17)	-3.4(13)	3.4(13)	19.0(13)
C14	71.1(19)	44.7(14)	37.6(14)	-4.8(11)	1.1(13)	-18.9(13)
C12	46.7(15)	49.6(15)	66.3(19)	11.0(13)	2.1(13)	-15.1(13)
C10	66.0(18)	49.7(15)	38.4(14)	8.6(11)	-1.3(12)	19.3(13)
N1	38.5(11)	32.6(9)	29.9(10)	-3.2(7)	-1.3(8)	6.2(8)
C2	42.7(13)	35.2(11)	30.2(12)	0.2(9)	-0.8(10)	1.5(10)
C7	39.7(13)	43.9(13)	47.3(15)	-11.8(11)	-1.6(11)	-1.8(11)
C4	44.1(13)	41.7(12)	34.4(12)	2.2(10)	9.5(10)	5.6(11)
C1	50.5(15)	40.6(13)	41.5(14)	1.2(10)	-5.4(11)	-7.4(11)
C3	40.5(14)	53.7(15)	53.7(16)	1.8(13)	12.0(12)	11.7(12)
C5	56.6(16)	42.0(13)	43.8(15)	-5.0(11)	-7.5(12)	20.8(12)
C6	60.4(18)	65.1(18)	45.4(16)	-2.1(13)	-11.8(13)	22.2(15)
C8	39.4(15)	89(2)	72(2)	-23.1(17)	8.4(14)	-19.1(15)
Te2	28.49(10)	21.97(9)	27.95(10)	-0.44(7)	1.25(7)	-0.36(7)
Te1	28.35(10)	26.30(9)	29.39(10)	1.06(7)	2.21(8)	-0.06(7)
Cl5	44.8(3)	35.7(3)	31.2(3)	-2.0(2)	5.9(2)	-0.9(2)
Cl2	56.3(4)	29.9(3)	50.4(4)	4.1(2)	2.5(3)	4.5(3)
Cl3	32	52	48	-9	2	-4
Cl1	44	43	32	1	4	-1
Cl4	33	36	47	3	-4	1
Cl6	45	26	45	-1	1	-2

Table S4. Bond Lengths for $(C_8H_{20}N)_2TeCl_6$.

Atom	Atom	Length/Å	Atom	Atom	Length/Å
N2	C15	1.524(3)	C4	C3	1.503(3)
N2	C11	1.527(3)	C5	C6	1.516(3)
N2	C9	1.515(3)	Te2	Cl5	2.5432(7)
N2	C13	1.514(3)	Te2	Cl5[1]	2.5432(7)
C15	C16	1.511(3)	Te2	Cl4	2.5346
C11	C12	1.508(3)	Te2	Cl4[1]	2.5346
C9	C10	1.522(3)	Te2	Cl6	2.5564
C13	C14	1.519(3)	Te2	Cl6[1]	2.5565
N1	C2	1.517(3)	Te1	Cl2	2.5473(6)
N1	C7	1.519(3)	Te1	Cl2[2]	2.5472(6)
N1	C4	1.525(3)	Te1	Cl3	2.5408
N1	C5	1.508(3)	Te1	Cl3[2]	2.5408
C2	C1	1.513(3)	Te1	Cl1[2]	2.5360
C7	C8	1.514(3)	Te1	Cl1	2.5360

Table S5. Bond Angles for $(C_8H_{20}N)_2TeCl_6$.

Atom	Atom	Atom	Angle/°	Atom	Atom	Atom	Angle/°
C15	N2	C11	111.96(18)	Cl4	Te2	Cl5	88.06(2)
C9	N2	C15	108.78(15)	Cl4	Te2	Cl5[1]	91.94(2)
C9	N2	C11	108.43(16)	Cl4[1]	Te2	Cl5	91.94(2)
C13	N2	C15	108.14(16)	Cl4[1]	Te2	Cl5[1]	88.06(2)
C13	N2	C11	108.58(15)	Cl4	Te2	Cl4[1]	180.0
C13	N2	C9	110.97(18)	Cl4[1]	Te2	Cl6	90.6
C16	C15	N2	115.43(19)	Cl4	Te2	Cl6[1]	90.6
C12	C11	N2	115.25(19)	Cl4	Te2	Cl6	89.4
N2	C9	C10	114.50(19)	Cl4[1]	Te2	Cl6[1]	89.4

N2	C13	C14	114.50(19)	Cl6	Te2	Cl6^{1}	180.0
C2	N1	C7	107.39(17)	Cl2^{2}	Te1	Cl2	180.0
C2	N1	C4	112.36(17)	Cl3^{2}	Te1	Cl2^{2}	90.209(15)
C7	N1	C4	108.83(17)	Cl3	Te1	Cl2	90.208(15)
C5	N1	C2	108.20(17)	Cl3^{2}	Te1	Cl2	89.791(16)
C5	N1	C7	111.90(18)	Cl3	Te1	Cl2^{2}	89.792(15)
C5	N1	C4	108.21(18)	Cl3^{2}	Te1	Cl3	180.0
C1	C2	N1	115.11(18)	Cl1^{2}	Te1	Cl2^{2}	88.186(14)
C8	C7	N1	115.6(2)	Cl1	Te1	Cl2	88.187(15)
C3	C4	N1	115.39(19)	Cl1	Te1	Cl2^{2}	91.814(14)
N1	C5	C6	115.4(2)	Cl1^{2}	Te1	Cl2	91.812(15)
Cl5	Te2	Cl5^{1}	180.0	Cl1	Te1	Cl3	91.0
Cl5	Te2	Cl6	89.548(12)	Cl1^{2}	Te1	Cl3	89.0
Cl5^{1}	Te2	Cl6	90.453(12)	Cl1^{2}	Te1	Cl3^{2}	91.0
Cl5^{1}	Te2	Cl6^{1}	89.546(12)	Cl1	Te1	Cl3^{2}	89.0
Cl5	Te2	Cl6^{1}	90.453(12)	Cl1	Te1	Cl1^{2}	180.0

Table S6. Torsion Angles for $(C_8H_{20}N)_2TeCl_6$.

A	B	C	D	Angle/°	A	B	C	D	Angle/°
C15	N2	C11	C12	-54.5(2)	C2	N1	C7	C8	-64.0(3)
C15	N2	C9	C10	-65.4(2)	C2	N1	C4	C3	-55.8(3)
C15	N2	C13	C14	173.85(19)	C2	N1	C5	C6	174.4(2)
C11	N2	C15	C16	-52.6(2)	C7	N1	C2	C1	173.91(19)
C11	N2	C9	C10	172.7(2)	C7	N1	C4	C3	63.0(3)
C11	N2	C13	C14	-64.5(2)	C7	N1	C5	C6	56.3(3)
C9	N2	C15	C16	-172.5(2)	C4	N1	C2	C1	-54.3(3)
C9	N2	C11	C12	65.5(2)	C4	N1	C7	C8	174.2(2)
C9	N2	C13	C14	54.6(2)	C4	N1	C5	C6	-63.6(3)
C13	N2	C15	C16	66.9(2)	C5	N1	C2	C1	65.1(2)
C13	N2	C11	C12	-173.8(2)	C5	N1	C7	C8	54.6(3)
C13	N2	C9	C10	53.5(2)	C5	N1	C4	C3	-175.2(2)
C15	N2	C11	C12	-54.5(2)	C2	N1	C7	C8	-64.0(3)
C15	N2	C9	C10	-65.4(2)	C2	N1	C4	C3	-55.8(3)
C15	N2	C13	C14	173.85(19)	C2	N1	C5	C6	174.4(2)
C11	N2	C15	C16	-52.6(2)	C7	N1	C2	C1	173.91(19)
C11	N2	C9	C10	172.7(2)	C7	N1	C4	C3	63.0(3)
C11	N2	C13	C14	-64.5(2)	C7	N1	C5	C6	56.3(3)
C9	N2	C15	C16	-172.5(2)	C4	N1	C2	C1	-54.3(3)
C9	N2	C11	C12	65.5(2)	C4	N1	C7	C8	174.2(2)

Table S7. Hydrogen Atom Coordinates (Å × 10^4) and Isotropic Displacement Parameters (Å^2 × 10^3) for $(C_8H_{20}N)_2TeCl_6$.

Atom	*x*	*y*	*z*	U_{eq}
H15A	7248.85	1526.04	1314.77	45
H15B	8077.21	2241.01	1072.03	45
H11A	7714.33	1527.13	3703.56	45
H11B	6912.96	2267.94	3954.86	45
H9A	6399.44	3343.62	2757.13	47
H9B	6154.67	2615.1	1882.04	47
H13A	8578.11	3333.73	2240.15	45
H13B	8825.36	2604.53	3113.72	45
H16A	8718.93	780.35	1245.45	78
H16B	8286.98	694.39	2377.5	78
H16C	9117.19	1415.29	2149.39	78
H14A	7608.27	4145.78	3393.58	77

H14B	8656.8	4045.78	3860.85	77
H14C	7815.92	3403.32	4267.94	77
H12A	6627.43	735.05	2647.81	81
H12B	5824.37	1470.37	2925.46	81
H12C	6239.53	812.17	3799.09	81
H10A	7197.42	3406.51	743.26	77
H10B	7377.56	4155.85	1617.3	77
H10C	6341.75	4045.23	1120.89	77
H2A	1767.58	2863.01	1036.84	43
H2B	2606.44	2157.13	800.97	43
H7A	2665.94	3762.12	2134.42	52
H7B	3238.36	3351.33	3087.44	52
H4A	1243.78	1936.57	3144.7	48
H4B	1900.65	2604.77	3806.5	48
H1A	1140.15	1501.03	384.12	66
H1B	774.81	1643.73	1536.12	66
H1C	1615.67	935.68	1302.79	66
H3A	630.61	3185.47	2240.99	74
H3B	1306.31	3862.37	2874.49	74
H3C	469.2	3323.9	3446.81	74
H5A	3449.84	1420.85	2091.75	57
H5B	2520.38	1012.3	2626.14	57
H6A	3875.86	2104.66	3695.18	85
H6B	3850.27	1011.56	3747.38	85
H6C	2988.1	1595.58	4209.71	85
H8A	3764.91	3112.39	995.08	100
H8B	4327.52	2661.61	1936.16	100
H8C	4308.5	3749.48	1804.15	100

Table S8. PL lifetimes of $(C_8H_{20}N)_2TeCl_6$.

λ_{ex} (nm)	λ_{em} (nm)	PL lifetime (ns)
405	540	223.80
405	550	225.40
405	560	230.01
405	570	228.94
405	580	232.49
405	590	229.80
405	600	236.43
405	610	234.74
405	620	237.18
405	630	236.95
405	640	237.71
405	650	236.23
405	660	237.24

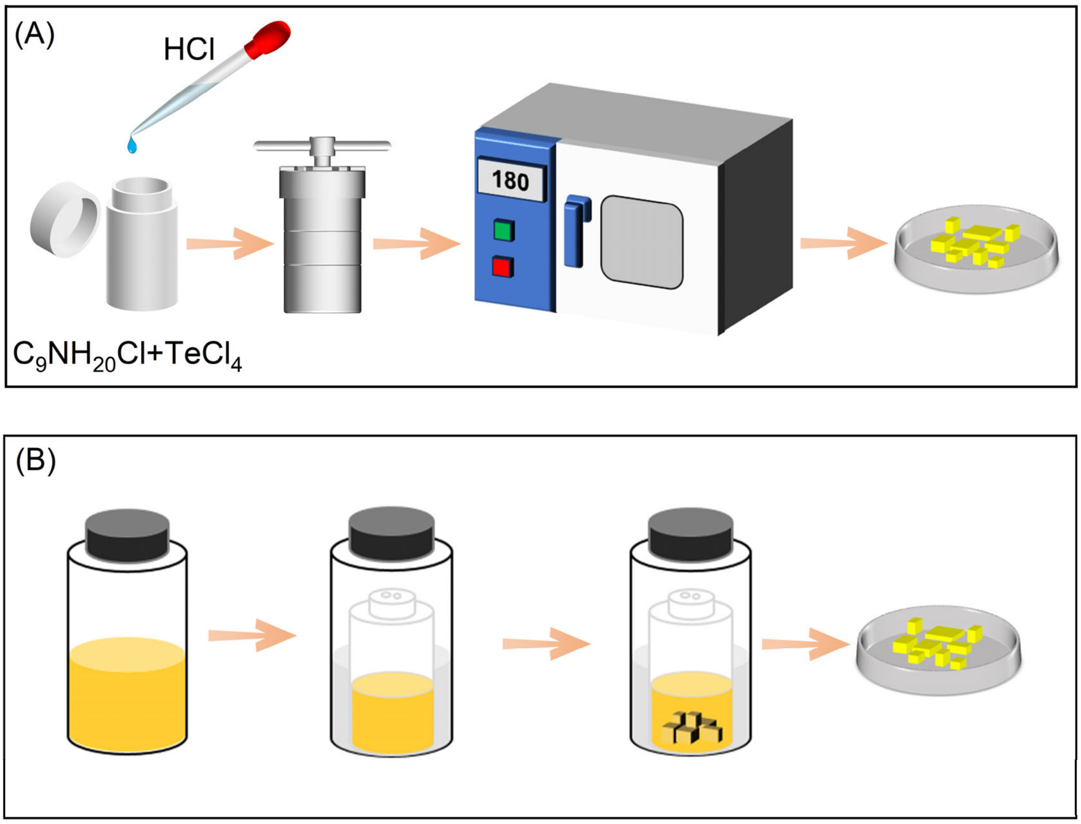

Figure S1. Diagram of synthesis of $(C_8H_{20}N)_2TeCl_6$ single crystals.

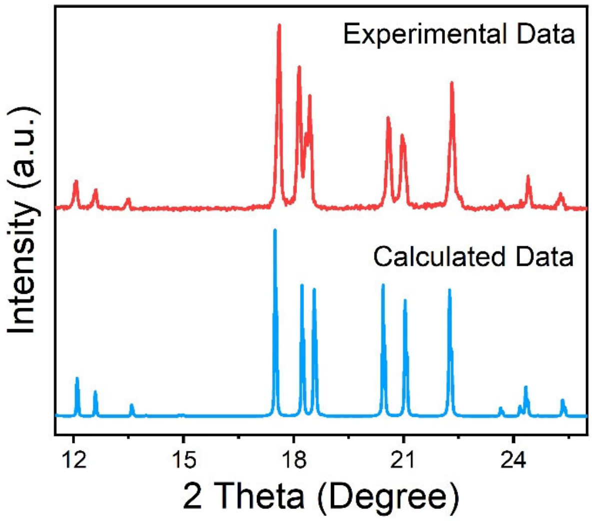

Figure S2. PXRD patterns of $(C_8H_{20}N)_2TeCl_6$ as well as its simulated result.

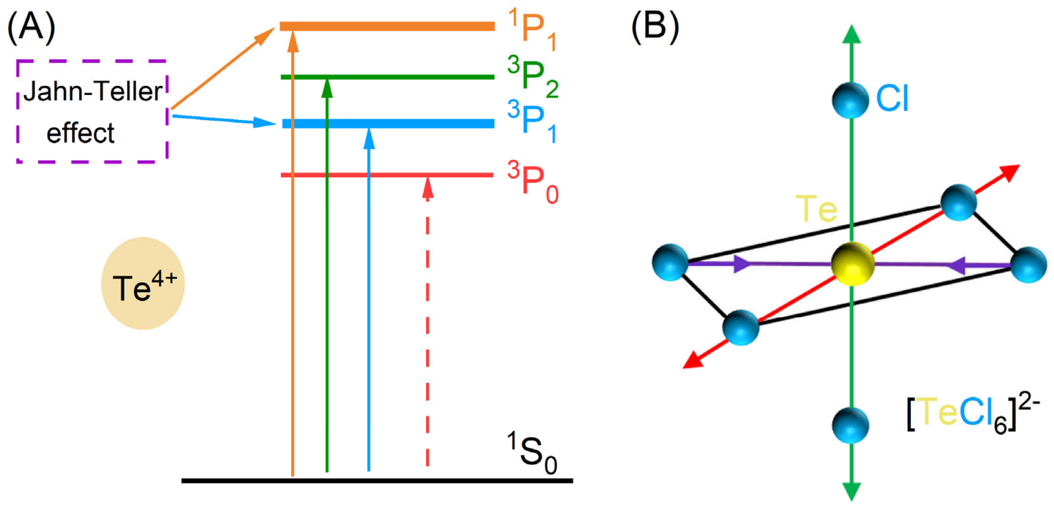

Figure S3. (a) Scheme of the energy level of $(C_8H_{20}N)_2TeCl_6$. **(b)** The *v*2 vibration mode of $[TeCl_6]^{2-}$ octahedra.

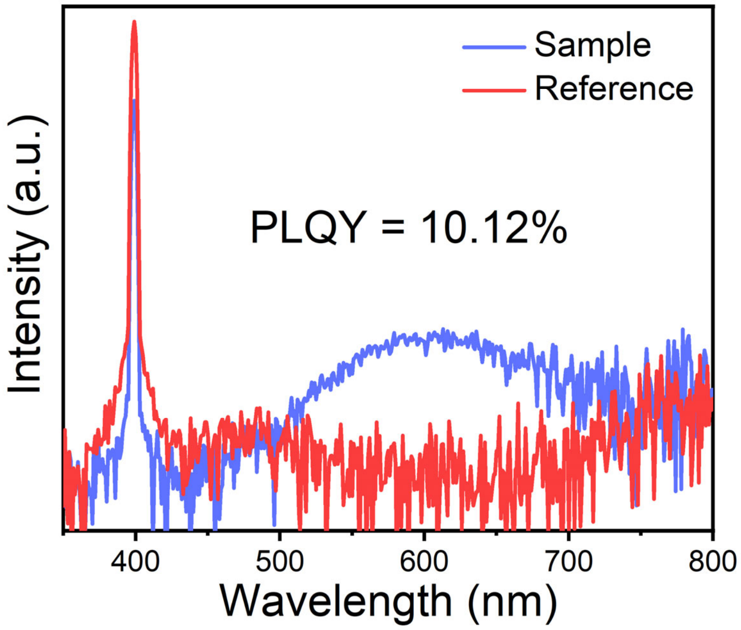

Figure S4. PLQY of $(C_8H_{20}N)_2TeCl_6$ at room temperature.
